# Design of a Broadband Solar Thermal Absorber Using a Deep Neural Network and Experimental Demonstration of Its Performance

**DOI:** 10.1038/s41598-019-51407-2

**Published:** 2019-10-21

**Authors:** Junyong Seo, Pil-Hoon Jung, Mingeon Kim, Sounghyeok Yang, Ikjin Lee, Jungchul Lee, Heon Lee, Bong Jae Lee

**Affiliations:** 1 Department of Mechanical Engineering, Korea Advanced Institute of Science and Technology, Daejeon, 34141 South Korea; 2 Department of Materials Science and Engineering, Korea University, 145 Anam-ro, Sungbuk-gu, Seoul 02841 South Korea

**Keywords:** Optics and photonics, Optical materials and structures

## Abstract

In using nanostructures to design solar thermal absorbers, computational methods, such as rigorous coupled-wave analysis and the finite-difference time-domain method, are often employed to simulate light-structure interactions in the solar spectrum. However, those methods require heavy computational resources and CPU time. In this study, using a state-of-the-art modeling technique, i.e., deep learning, we demonstrate significant reduction of computational costs during the optimization processes. To minimize the number of samples obtained by actual simulation, only regulated amounts are prepared and used as a data set to train the deep neural network (DNN) model. Convergence of the constructed DNN model is carefully examined. Moreover, several analyses utilizing an evolutionary algorithm, which require a remarkable number of performance calculations, are performed using the trained DNN model. We show that deep learning effectively reduces the actual simulation counts compared to the case of a design process without a neural network model. Finally, the proposed solar thermal absorber is fabricated and its absorption performance is characterized.

## Introduction

In recent decades, clean and abundant solar energy has been considered to be a promising renewable energy source to help mitigate rising fossil fuel prices, global warming, and environmental pollution. One of the efficient ways to utilize solar energy is to use a solar thermal energy conversion system. Compared to conventional photovoltaics, the advantage of solar thermal energy conversion is the achievement of high energy conversion efficiency via broad-band absorption across the entire solar spectrum. To obtain the maximum conversion efficiency of solar radiation, the solar thermal absorber should be able to interact with a broad spectrum of solar radiation from the visible to the near-infrared (IR) spectral region, while suppressing mid-IR emission to minimize heat loss at high temperatures. However, it remains challenging to achieve a highly efficient solar absorber because of limitations in the fabrication of complicated nanostructures. To overcome this fabrication difficulty, we hereby propose a relatively simple subwavelength-sized nanostructure and perform design optimization to achieve high solar thermal conversion efficiency.

By inducing electromagnetic resonance, subwavelength-sized nanostructures have great potential for use in tailoring the radiative properties of system, especially the absorption spectrum^1–4^. Well-designed nanostructures with desirable radiative properties can be utilized as selective^1,3,4^ and broadband absorber^5^. In designing subwavelength-sized nanostructures, precise prediction of the corresponding radiative properties resulting from light-structure interactions is crucial. Computational electrodynamics methods, such as rigorous coupled-wave analysis^6^ (RCWA) or the finite-difference time-domain^7^ (FDTD) method, are often employed for this purpose. Because the optical resonance phenomena occurring with periodic nanostructures are complicated and highly correlated with the structural geometry, determining the structure that possesses the desired radiative properties cannot be accomplished via simple parametric studies. Rather, more systematic structural optimization and parametric analysis are needed.

Recently, design and analysis methods that do not consider input and output configurations have been studied^8,9^. Because these methods treat a problem as a black box, they are beneficial in that any problems can be investigated. Furthermore, problems can be solved globally instead of locally because the methods are applicable over entire problem domains. For instance, search based algorithms^10^ (simulated annealing, genetic algorithm, particle swarm optimization, etc.) have been used for global optimization. To explore the whole variable space, the proposed approaches are based on numerous performance computations. To design subwavelength-sized nanostructures by applying the algorithms listed above, significant reduction of performance computation time is necessary. In this research, a surrogate model based on sample training is employed to reduce the computational cost of solar thermal absorber design.

A surrogate model can, based on training data, predict the behavior of an actual system. To construct a more precise surrogate model, machine learning has recently been used as a modeling technique^11–13^. In particular, deep learning, which is a machine learning method that uses a deep neural network (DNN) as a model, has been broadly applied to solving mechanical problems. DNNs have been applied for estimating certain properties and/or the performance of a system^11,12^, or for predicting the future behavior of a system^13^. For instance, Sajedian *et al*.^12^ predicted the resonant properties of a plasmonic metamaterial using a DNN; they also predicted the shape of a structure possessing an arbitrary absorption spectrum. Most works, however, are focused on precise output prediction with respect to input only, even though any network that makes good predictions can be used as a surrogate model. By considering the model as an alternative to an actual system, further investigation of the system, e.g., optimization can be conducted based on the model. In addition, because a surrogate model can reduce the computational cost by directly predicting an output from an input, analysis methods requiring heavy computational resources can also be applied.

In the present study, we demonstrate first a surrogate design process utilizing a surrogate DNN model to reduce the time required for estimating the radiative properties of subwavelength-sized nanostructures. As an example, we propose a new design for a broadband solar thermal absorber based on a simple two-dimensional grating^14^. After successful training, Various optimizations and sensitivity analysis will be conducted with the trained network model replacing the original RCWA calculations. For optimization, particle swarm optimization^15^ (PSO) and multi-objective optimization^16^ (MOO) methods will be employed to find deterministic, robust^17^, and constrained optima. Furthermore, we will also fabricate the broadband solar thermal absorber, made of an embedded Cr grating, by nanoimprint lithography (NIL) and measure its solar absorptance.

## Modeling

**Figure 1:**
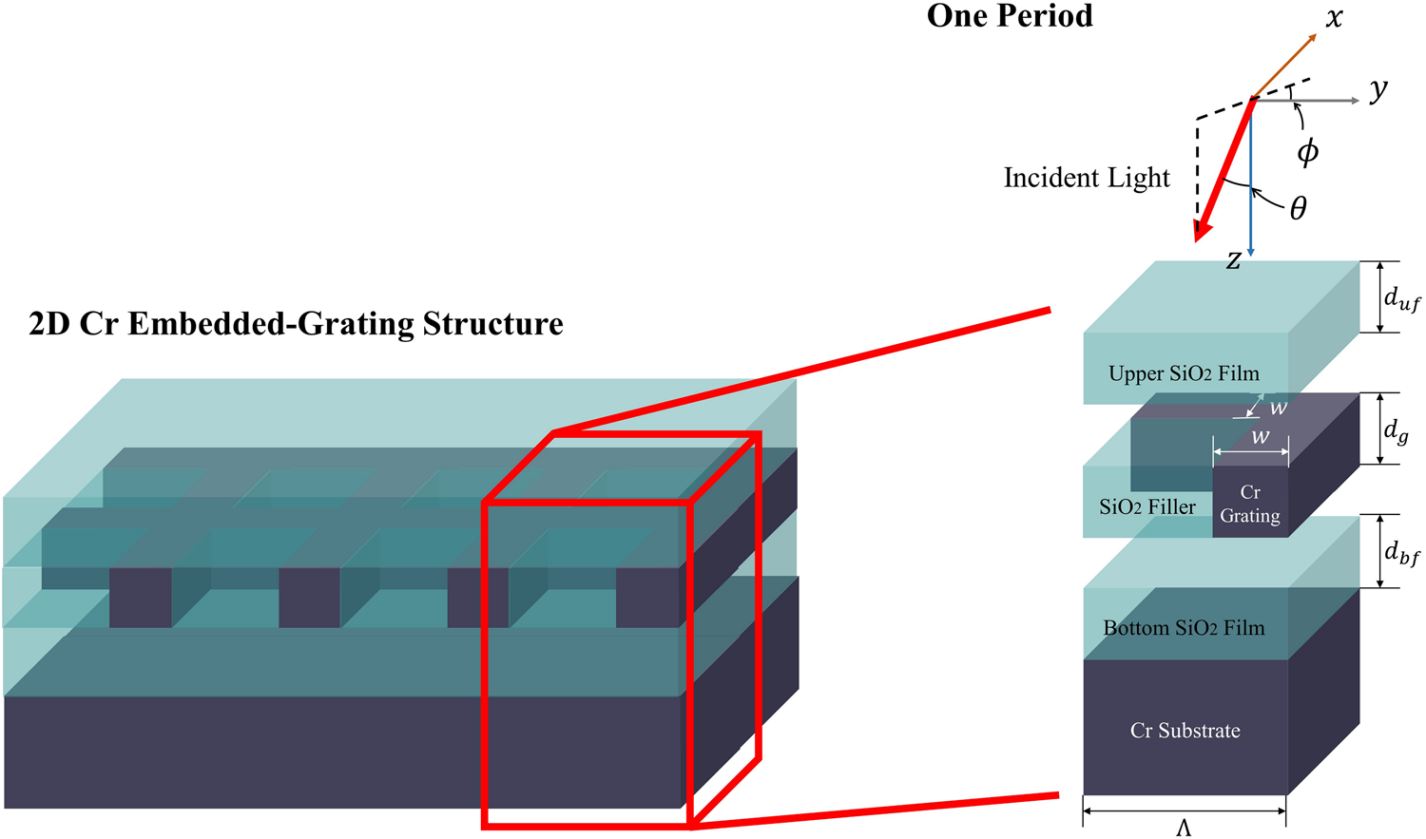
Schematic of a Cr embedded-grating solar absorber.

Because the solar spectrum covers from 280 nm to 4,000 nm, a broadband absorber is necessary to fully utilize solar energy. Although complicated nanostructures have been proposed as efficient solar thermal absorbers^2,18,19^, a simple fishnet design is proposed here to simplify the fabrication process while maintaining high solar absorption performance. As an absorbing material, we chose Cr because it has stronger intrinsic absorption than many novel metals and can also support plasmonic resonance in the visible spectrum^20^. As shown in Fig. 1, the proposed structure includes a fishnet grating (made of Cr), which is embedded in a SiO_2_ layer. The topmost SiO_2_ layer is intentionally added to prevent physical damage of the grating structure, as well as to induce an anti-reflection effect^21^. In Fig. 1, the dimensional parameters (i.e., period Λ, grating width *w*, grating thickness *d*_*g*_, thickness of topmost SiO_2_ layer *d*_*uf*_, and thickness of bottom SiO_2_ film *d*_*bf*_) and polar (*ϕ*) and azimuthal angles (*θ*) of the incident radiation are depicted. As a reference, based on the results reported in our earlier publication^14^, we set Λ = 600 nm, *w* = 50 nm, *d*_*g*_ = 200 nm, *d*_*uf*_ = 100 nm, and *d*_*bf*_ = 100 nm. For simplicity, both *ϕ* and *θ* are set to zero. Since the grating structure is symmetric along both the *x*- and *y*-axes, only the transverse magnetic (TM) polarization, in which the electric field oscillates in the *x*-direction, is considered. The open-source RCWA software package^22^ is employed to calculate the spectral absorptance *α*_*λ*_. With respect to the optical constants of the constitutive materials, the Lorentz-Drude model^20^ is used for Cr, and tabulated data^23^ are used for SiO_2_. Using the spectral absorptance, the solar absorptance *α*_*sol*_ is calculated as

1 $${\alpha }_{sol}=\frac{{\int }_{300\,{\rm{nm}}}^{2500\,{\rm{nm}}}{\alpha }_{\lambda }{G}_{dsi}(\lambda )d\lambda }{{\int }_{300\,{\rm{nm}}}^{2500\,{\rm{nm}}}{G}_{dsi}(\lambda )d\lambda }$$

where *G*_*dsi*_(*λ*) is the direct solar irradiance spectrum. A total of 221 data points (each with 10-nm intervals) are calculated in the wavelength region of 300 nm to 2,500 nm, where approximately 99% of solar energy is radiated. The RCWA code was executed using a workstation with an Intel Xeon processor (eight cores with 2.70 GHz); it took approximately 20 min to obtain each *α*_*λ*_ spectrum.

To construct a surrogate model that can predict the solar absorptance of Cr embedded-grating structures with various geometric parameters, a DNN was employed. Because the effects of the number and size of hidden layers are not clearly known^24^, various numbers of nodes and layers of a DNN structure were studied. To achieve high accuracy, the DNN structure was constructed as complicated as possible because the functional relationship between the input and output variables cannot be known *a priori*. To train the DNN model, a training data set, i.e., {Input:(Λ, *w*, *d*_*g*_, *d*_*uf*_, *d*_*bf*_)→Output:*α*_*sol*_}, was first generated. To achieve unbiased sampling, a Latin-Hypercube (LH) sampling^25^ spread within the parametric boundaries (Table 1) was adopted. Training of the DNN model was started with 300 training data set. A validation accuracy of the DNN model was measured by the root mean square error (RMSE) between the true *α*_*sol*_ (i.e., directly obtained from the RCWA calculation) and the ${\tilde{\alpha }}_{sol}$ predicted from the DNN model of additional 100 LH samples, which were not included in the training data set. If the calculated RMSE did not reach a certain criterion (set to be 0.01 in this work), we added more samples to the training data set. At each step of validation, from 50 to 100 samples were added to the training data set until the convergence criterion is satisfied. In addition to the validation data set, we also compared the predicted optimum value with validated optimum value to make sure that the DNN model captures response of the system in the extrema. As a result, we achieved the RMSE of 0.003 with a total of 1,566 training data set. Furthermore, a regularization process^26^ along with the validation was also applied to prevent the over-fitting effect of an excessively complex model. In the present study, we designed a structure of DNN as five hidden layers with ten nodes in each layer. All of the nodes are fully connected, and it took approximately a minute to train our DNN model. As we aimed earlier, the DNN model had lower validation error than the other surrogate modeling methods (see Supplementary information, Figure S1).

**Table 1:** Range of dimensional variables.

Parameter	Λ (nm)	*w* (nm)	*d*_*g*_ (nm)	*d*_*uf*_ (nm)	*d*_*bf*_ (nm)
Lower Bound	300	50	5	50	50
Upper Bound	600	Λ - 50	200	150	150

For the deterministic and constrained optimization, particle swarm optimization (PSO)^15^, which utilizes swarm intelligence, was applied over the network to determine the globally optimal design of the solar absorber. Because LH samples are distributed uniformly inside the variable range, over/underestimation problems occurred because of the lack of samples near the boundaries. To resolve this issue, additional data on near-boundary samples should be included in the training data set. As listed in Table 2, an additional 288 near-boundary samples were trained. In addition to the deterministic optimization, we also conducted robust optimization^17^ to find an absorber design that was relatively insensitive to fabrication errors. A multi-objective genetic algorithm^16^ was used for the robust optimization^19^; that is, the one objective function was to maximize the solar absorptance, and the other was to minimize the variation of the solar absorptance (i.e., standard deviation *σ*_*sol*_) arising because of fabrication errors. In this work, the fabrication errors were assumed to follow a Gaussian distribution within the following uncertainty ranges^27^: 50 nm for Λ, 25 nm for *w*, 5 nm for *d*_*g*_, and 10 nm for both *d*_*uf*_ and *d*_*bf*_. Please note that the optimization algorithms employed here (i.e., PSO and GA) explore the continuous variable space using vectorized searching rules^15,16^. After each iteration (or generation for GA) of the algorithm, optimization process will be terminated by a certain termination criterion. Here, our optimization algorithms stop when the optimal value stalled at least subsequent 50 iterations (or generations for GA).

**Table 2:** Near-boundary dimensions for samples to overcome divergence problems.

Parameter	Near-boundary Dimension (nm)
Λ	300/400/500/600
*w*	50/(Λ−50)
*d*_*g*_	5/10, 195/200
*d*_*uf*_	50/100/150
*d*_*bf*_	50/100/150

## Results and Discussion

First, we compare the solar absorptance of the reference design and the deterministic optima. One can simply regard the reference design as an arbitrary case because its geometry was simply adopted from an earlier work (i.e., Λ = 600 nm, *w* = 50 nm, *d*_*g*_ = 200 nm, *d*_*uf*_ = 100 nm, and *d*_*bf*_ = 100 nm)^14^ without carrying out an optimization process. As shown in Table 3, the deterministic optimization boosts up the solar absorptance value to approximately 0.95. The difference between the value predicted by the DNN model (i.e., ${\tilde{\alpha }}_{sol}$) and the exact value obtained by RCWA calculation (i.e., *α*_*sol*_) is only 0.002, indicating the excellent convergence of the surrogate model.

**Table 3:** Optimization results for the DNN model trained with 1566 data set. ${\tilde{\alpha }}_{sol}$ is the solar absorptance predicted by DNN. ${\alpha }_{sol}$ is the solar absorptance calculated by RCWA for validation. ${\sigma }_{sol}$ is the standard deviation of the distributed ${\tilde{\alpha }}_{sol}$ due to fabrication uncertainty of each dimensional variables.

Category	Λ (nm)	*w* (nm)	*d*_*g*_ (nm)	*d*_*uf*_ (nm)	*d*_*bf*_(nm)	${\tilde{{\boldsymbol{\alpha }}}}_{{\boldsymbol{sol}}}$	*α*_*sol*_	*σ*_*sol*_
Reference design	600	50	200	100	100	0.819	0.817	0.029
Deterministic optimum	330	161	9	94	91	0.949	0.947	0.057
Robust optimum	426	88	190	92	113	0.919	0.918	0.017
Constrained optimum	600	300	8	91	95	0.916	0.918	0.051

**Figure 2:**
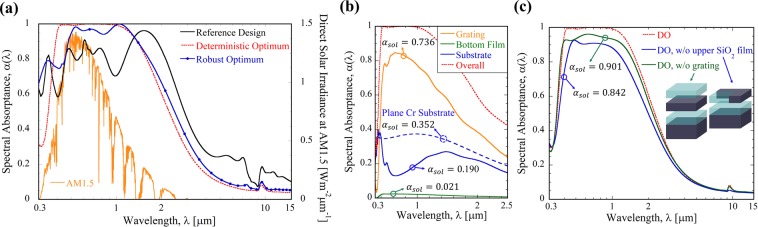
(**a**) Absorption spectra of reference design (black), deterministic optimum (red dotted), and robust optimum structures (blue with circle); (**b**) Separate contribution of each layer in the deterministic optimum (orange for grating, blue for Cr substrate, and green for bottom SiO_2_ film layer) compared to that of a planar Cr substrate (blue dotted); and (**c**) Effect of the upper SiO_2_ film (blue) or Cr grating (green) in the deterministic optimum. The open-source RCWA software package^22^ was used for calculation of spectral absortance.

Figure 2(a) shows the calculated spectral absorptance in the solar spectrum as well as in the mid-IR spectral region. It is clear that the particle swarm optimization (i.e., deterministic optimum) results in near-unity spectral absorptance in a range from 400 nm to 1,000 nm, where a large portion of the solar irradiance is located. The corresponding absorptance spectrum of the deterministic optimum matches well with that of the solar spectrum. The spectrum indicated as robust optimum will be discussed later. It is interesting to note that only 9-nm-thick Cr grating can yield such a high *α*_*sol*_ value in the deterministic optimum. Recall that the DNN model is the most accurate one among other considered surrogate models, as noted in Figure S1 of Supplementary Information. Additionally, the DNN model can predict the performance of solar absorber in any points on the continuous variable space (i.e., five-dimensional space with Λ, *w*, *d*_*g*_, *d*_*uf*_, and *d*_*bf*_). Therefore, we can find the “global” optimization point within the design space defined by Table 1 not simply selecting the best-performing configuration out of the training data set.

In Fig. 2(b), the absorption contribution of each layer in the deterministic optimum is further analyzed. Here, the absorption of the upper SiO_2_ layer is not shown in the figure because it is negligible. It can be clearly seen from the figure that the 9-nm-thick Cr grating indeed dominantly contributes (i.e., 0.736) to the solar absorptance; that is, about 78% of the total absorption takes place inside the grating region. Note that total solar absorption of the deterministic optimum (i.e., *α*_*sol*_ = 0.947) can be obtained simply by summing all contributions of each layers; that is, *α*_*sol*_ = 0.736(grating) + 0.021(bottom SiO_2 _film) + 0.190(Cr substrate) = 0.947.

To elucidate the mechanisms of absorption enhancement, we investigate the effects of the topmost SiO_2_ layer as well as the Cr grating in the deterministic optimum. As shown in Fig. 2(c), the upper SiO_2_ layer certainly plays a role in enhancing the solar absorptance. As mentioned earlier, the upper SiO_2_ layer is expected to lead to a gradual increase in the refractive indices and cause an anti-reflection effect^21^, boosting the solar absorptance value from 0.842 to 0.947. If there is no Cr grating pattern (i.e., replacing the Cr grating by a smooth 9-nm Cr thin film, *w* = Λ), the resulting absorptance spectrum exhibits slightly lower performance (i.e., approximately 95% of the deterministic optimum), especially in the wavelength region from 400 nm to 1,000 nm. In terms of the solar absorptance, the Cr thin-film structure results in *α*_*sol*_ = 0.901, which is 5% smaller than that of the deterministic optimum. Notice that the Cr thin-film structure is nothing but a Fabry-Pérot structure made of a refractory metal^28^, which can also exhibit an excellent absorption performance in the solar spectrum. By opening about 21% of the surface area in the Cr film (i.e., [1 − *w*/Λ]^2^), we can achieve a 5% enhancement of the solar absorptance, suggesting that the enhanced absorption by employing the Cr pattern is due to the near-field interaction of diffracted evanescent waves with the subwavelength-sized nanostructures^5,14^.

We now consider the potential performance degradation of a real sample caused by inevitable fabrication errors. In Table 3, *σ*_*sol*_ indicates the standard deviation of ${\tilde{\alpha }}_{sol}$ at the given design point as a result of random variations in the dimensional variables. At the deterministic optimum point, where the corresponding ${\tilde{\alpha }}_{sol}=0.949$, the resulting *σ*_*sol*_ = 0.057, which is approximately 6% of the ${\tilde{\alpha }}_{sol}$ value. At the robust optimum point, the solar absorptance is slightly reduced to ${\tilde{\alpha }}_{sol}=0.919$, but its standard deviation becomes *σ*_*sol*_ = 0.017, which is only 1.8% of the ${\tilde{\alpha }}_{sol}$ value and comparably lower than one of the deterministic optimum. It can be seen from Fig. 2(a) that the robust optimum weakens primarily the short-wavelength absorptance (less than 500 nm). Nevertheless, by compromising the solar absorptance by 3%, we can come up with a nanostructure that is substantially less sensitive to the variations of design variables.

**Figure 3:**
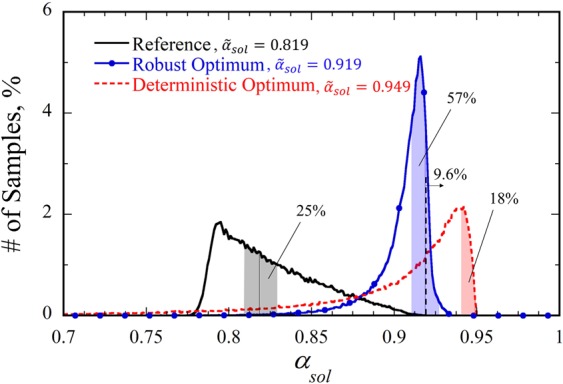
Population histogram of the solar absorptance at deterministic and robust optima. For comparison, population density for the reference design is also shown.

To quantify the robustness, the probability density distribution of the solar absorptance is plotted in Fig. 3. The region of ${\tilde{\alpha }}_{sol,opt}\pm 0.01$ is compared for the deterministic and robust optima. Although only 18% of samples are included in the absorptance range between 0.939 and 0.959 for the deterministic optimum, the robust optimum includes 57% of the samples in the range of $0.909 < {\tilde{\alpha }}_{sol} < 0.929$. Since the deterministic optimum obtained from PSO corresponds to the global optimum, fabrication errors will always deteriorate the performance. For the robust optimum, however, 9.6% of the fabricated samples can show better absorptance values than the target value (i.e., ${\tilde{\alpha }}_{sol}=0.919$). Although it is not shown here (see Supplementary Information, Figure S2), we also have employed Monte Carlo-based global sensitivity analysis^29^ to determine a solar absorptance is the most sensitive to which dimensional variable. The grating width (*w*) is found to be the most affecting, and the thickness of the grating (*d*_*g*_) is the second most affecting factor. This is the reason why the *w* and *d*_*g*_ values of the robust optimum vary widely from those of the deterministic optimum (refer to Table 3).

For the deterministic optimization process using PSO, a swarm comprising 50 individuals communicated with each other, and their fitness values (i.e. ${\tilde{\alpha }}_{sol}$) were compared. As a result, approximately 60 iterations took place during the optimization process (i.e., the DNN estimation occurred about 3,000 times on average). Multi-objective optimization for the robust optimization process to compare the multiple fitness values was more complicated. To compute one *σ*_*sol*_, 100,000 estimations of ${\tilde{\alpha }}_{sol}$ around the target point were necessary. Moreover, about 100 iterations were needed to find the multiple solutions of the MOO process. In other words, approximately 500 million (i.e., 5,000 × (100,000 + 1)) estimations of ${\tilde{\alpha }}_{sol}$ were made to find the robust optimum. For the sensitivity analysis, Monte-Carlo estimation with 100,000 samples was required for each variable set. Therefore, a total of 3.2 million (i.e., 2^5^ × 100,000) DNN estimations were required. Although enormous numbers of performance estimation were required in the aforementioned processes, the entire calculation time was not overly long with the use of the surrogate model. By replacing the electrodynamic simulation of *α*_*sol*_ with the DNN estimation (i.e., ${\tilde{\alpha }}_{sol}$), the time for required for the performance estimation took less than 0.1 sec; the original RCWA simulation took approximately 20 min. Furthermore, the DNN is suitable for massive numbers of sample computations because it is based on the matrix representation. Thus, even though about 500 million estimations were required, the entire optimization process could be finished within an hour. Please refer to Figure S3 in Supplementary Information for more clear comparison between RCWA computation requirements.

## Experiments

Before fabricating the proposed solar absorber, a constrained optimization process was also conducted to optimize the structure under the physical limitations associated with fabrication, for example, patternable structures using available masks. In this work, Λ- and *w*-constrained optimization was performed as noted in Table 3. With fixed lateral dimensions of Λ = 600 nm and *w* = 300 nm, the performance can be maximized by changing other parameters (i.e., *d*_*g*_, *d*_*uf*_, and *d*_*bf*_). Since *w*, which was found to affect the solar absorptance most, is fixed now, the resulting optimum is expected to be less absorbing than the previously conducted deterministic optimum. Surprisingly, the constrained optimization resulted in the solar absorptance of ${\tilde{\alpha }}_{sol}=0.916$, which is lower by 3% only than the deterministic optimum although the number of dimensional variables is reduced from five to three. Note that the constrained optimum structure has two great advantages regarding fabrication. One is the ease of fabrication, as its lateral size is larger; the other is the lower fabrication cost, as the NIL mask for the pattern is already available (as imposed by the constraints).

**Figure 4:**
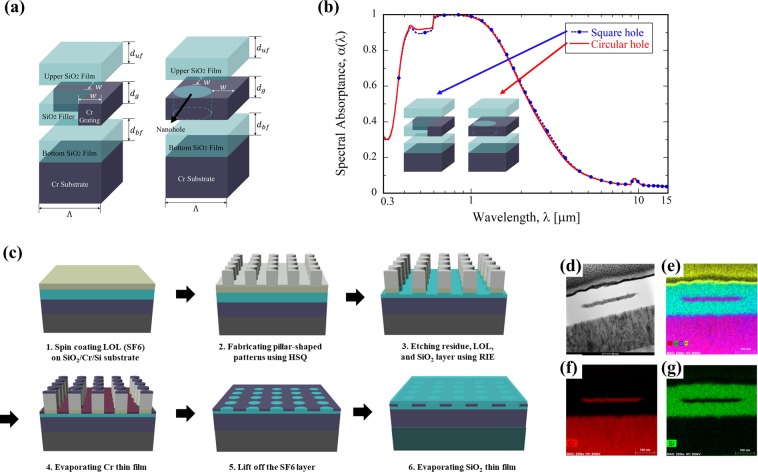
(**a**) Original square hole and alternative circular hole grating design; (**b**) Absorption spectra (calculated by the open-source RCWA software package^22^) of constrained optimum for proposed fishnet grating structure (blue with circle) and alternative nanodisk structure (red); (**c**) Fabrication processes of Cr embedded-grating solar absorber using NIL; (**d**) TEM-SEM image of fabricated Cr embedded-grating solar absorber structure; and (**e–g**) HAADF-STEM cross-sectional image and EDs elemental mapping image of Cr embedded-grating structures: Cr (red), Si (green), and O (blue).

As illustrated in Fig. 4(a), we further simplified the structure by changing the square hole in the Cr grating to a circular hole, which will greatly reduce the fabrication uncertainty. As can be seen from Fig. 4(b), the absorptance spectra of the two structures (one with a square hole and the other with a circular hole) are nearly the same. Thus, the Cr grating with a circular hole was fabricated as briefly summarized below [also refer to Fig. 4(c)]. First, Cr (300 nm) and SiO_2_ (100 nm) thin films were fabricated on a Si substrate using an e-beam evaporator and plasma-enhanced chemical vapor deposition (PECVD) equipment. A 200-nm-thick lift-off layer (LOL, PMGI SF6) was spin-coated on the SiO_2_/Cr-deposited Si substrate. Then, calculated pillar-shaped patterns were formed with hydrogen silsesquioxane (HSQ) material using a prepared polydimethylsiloxane (PDMS) mold and NIL technology. The PDMS mold was duplicated by a Si master stamp that was fabricated through a series of processes, including photolithography and reactive ion etching. Next, the residual HSQ layer was removed by reactive ion etching. After the deposited SiO_2_ was etched, Cr was evaporated, and the LOL was removed using dimethylformamide. Note that e-beam evaporation offers simplicity in the deposition process with excellent control of the deposition rates as low as 1 nm per minute^30^. Therefore, thickness of the pillar side can be precisely controlled. Finally, the SiO_2_ layer was deposited using PECVD equipment. The TEM image in Fig. 4(d) clearly indicates that the Cr grating pattern is embedded in the SiO_2_ film. It can be also concluded from the HAADF-STEM images in Fig. 4(e–g) that the Cr embedded-grating structure was successfully fabricated. We fabricated 2 × 2 cm^2^ samples for the spectroscopic measurements.

**Figure 5:**
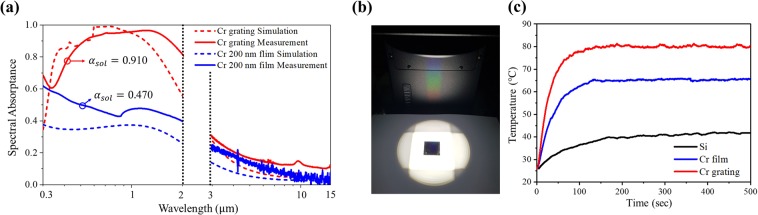
(**a**) Simulated spectral absorptance (by the open-source RCWA software package^22^) of Cr grating (red dotted) and plane 200-nm-thick Cr film (blue dotted), and measured spectral absorptance of Cr grating (red) and plane 200-nm-thick Cr film (blue); (**b**) Setup of the photo-thermal conversion experiment; and (**c**) Temperature changes of Si, 200-nm-thick Cr film and Cr grating during 600 sec under the one-sun condition.

Figure 5(a) shows a comparison of the spectral absorptance of the Cr grating structure and 200-nm-thick Cr film at room temperature. The radiation penetration depth of Cr is less than 10 nm in the solar spectrum; thus, the 200-nm-thick Cr film and fabricated grating structure can be treated as semi-infinite, opaque medium. Therefore, the spectral absorptance can be obtained from *α*_*λ*_ = 1 − *ρ*_*λ *_^21^. In this work, we have employed two spectrometers with an integrating sphere to measure the normal-hemispherical reflectance. A UV-VIS spectrometer (Shimadzu, UV-3600 plus) was used to measure the normal-hemispherical reflectance in the wavelength region from 0.3 *μ*m to 2.0 *μ*m, and a FT-IR spectrometer (ABB Bomem, FTLA 2000 series) is used in the wavelength region from 3 *μ*m to 15 *μ*m. Because of low signal-to-noise ratio of the FT-IR spectrometer, spectral absorptance cannot be measured between 2.0 and 3.0 *μ*m. The measured *α*_*sol*_ of the Cr-grating structure is nearly twice that of the planar Cr film. The calculated absorptance spectrum of the Cr-grating structure captures general features of the measured spectrum, but little discrepancy exists between the simulation and the experiment. As also noted from Fig. 5(a), the simulated and measured absorptance spectra of the plane Cr film show non-negligible differences in the solar spectrum. These differences can be attributed to the difference in optical constants for Cr between simulation and experiment.

Finally, we performed a photo-thermal conversion experiment to quantitatively compare the absorptance performance of fabricated samples. Here, we consider a plane Si wafer (approximately 500 *μ*m thick), a 200-nm-thick Cr film coated on a Si wafer, and the constrained optimum Cr grating structure fabricated on a Si wafer. The temperature increment rate of the substrate was measured using an IR camera (Fluke, TiS20) and a K-type thermocouple while samples were exposed to one-sun conditions (Mc Science, K201 Xe55), as shown in Fig. 5(b). It can be seen from Fig. 5(c) that the Cr grating structure reaches the highest temperature, 80.7ºC while the plane Si sample has the lowest temperature. With a solar absorptance of 47%, the 200-nm-thick Cr film can reach temperatures as high as 65.5ºC. The temperature difference between ambient conditions (25ºC) for the Cr grating structure is approximately 56ºC, while that of the Cr film is 41ºC. Theoretically, the temperature difference should be proportional to the energy input (i.e. solar absorptance, *α*_*sol*_). In this experiment, the ratio between temperature differences is 1.37 (56/41), while the ratio between solar absorption is 1.94 (0.910/0.470). That is, heat loss caused by conduction between the sample mounter and IR emission caused the steady-state temperature of the Cr grating structure to be lower than expected. Nevertheless, a structure with higher absorption should be designed since heat loss will be minimized in practical usage of solar thermal absorption system. For instance, evacuated space could be employed between absorbing surface and package surrounding it. With properly designed solar absorber system, the achievable temperature of the optimized structure will be proportionally high to its solar absorptance. Moreover, the time to reach the maximum temperature for the Cr grating absorber is the shortest because of its highest absorption, confirming its excellent absorption performance.

### Statistical analysis

From the learning procedure of networks to the optimization process, all calculations were performed with MATLAB global optimization toolbox.

## Conclusion

To summarize this study, deep learning, a state-of-the-art modeling technique, has been employed to accurately model the performance of subwavelength-sized nanostructures. Deep learning significantly enhances the scope of the investigation by reducing the overall calculation time. For example, recently, studies of performance computation-based analysis techniques have been carried out, such as search-based optimization (particle swarm optimization, genetic algorithm) and Monte Carlo estimation. These processes are practical and concise; however, they require enormous amounts of time for the performance computations regarding the overall solution space. It is impossible to apply these methods directly to our original problem because of the heavy computational cost. Deep learning successfully makes it possible using only 1,566 iterations of RCWA simulation, and an infinitesimal time for performance estimation. Moreover, the performance of the optimized result has been validated through actual fabrication and experiments. Hence, our new approach requires fewer performance calculations than methods without deep learning (i.e. 3,000 to 500 million RCWA computations were originally needed), while maintaining the estimation accuracy higher than other modeling techniques and being able to predict unknown performance beyond ordinary intuition.

With the constructed DNN model, we conducted three optimization processes: (1) deterministic optimization; (2) robust optimization; and (3) constrained optimization. The deterministic optimization process is to find the globally best performance (i.e. maximal *α*_*sol*_), which can be served as a reference for other two optimization processes. The robust optimization is to find the structure whose solar absorptance is close (i.e., slightly less) to the deterministic optimization but shows great robustness to the fabrication uncertainties. Finally, the constrained optimization is to find the best performance under the constraint on some of the design parameters, which is usually determined by considering fabrication feasibility (or fabrication easiness). The optimization methods introduced in this work using the data science techniques can be effectively employed for designing nanostructures with desirable spectral radiative properties.

## Supplementary information

Supplementary Information

## Data Availability

All data that support the findings of this study are available from the corresponding author upon request.
