# Supplementary material for: Design of a Broadband Solar Thermal Absorber Using a Deep Neural Network and Experimental Demonstration of Its Performance
Source: Sci Rep. 2019 Oct 21;9:15028. doi: 10.1038/s41598-019-51407-2 (PMC6803693; doi:10.1038/s41598-019-51407-2)
Supplement: Supplementary file 1 — Supplementary Information [file 41598_2019_51407_MOESM1_ESM.pdf]

Supplementary information: Design of a Broadband Solar Thermal Absorber Using a Deep Neural Network and Experimental Demonstration of Its Performance

Junyong Seo[1,†], Pil-Hoon Jung[2,†], Mingeon Kim[1], Sounghyeok Yang[1], Ikjin Lee[1], Jungchul Lee[1], Heon Lee[2,*], and Bong Jae Lee[1,*]

[1]Department of Mechanical Engineering, Korea Advanced Institute of Science and Technology, Daejeon 34141, South Korea
[2]Department of Materials Science and Engineering, Korea University, 5-1 Anam-dong, Sungbuk-gu, Seoul 02586, South Korea
[†]These authors contributed equally.
[*]Corresponding authors: heonlee@korea.ac.kr (H. Lee) & bongjae.lee@kaist.ac.kr (B.J. Lee)

1 Estimation accuracy comparison between other surrogate modelling methods

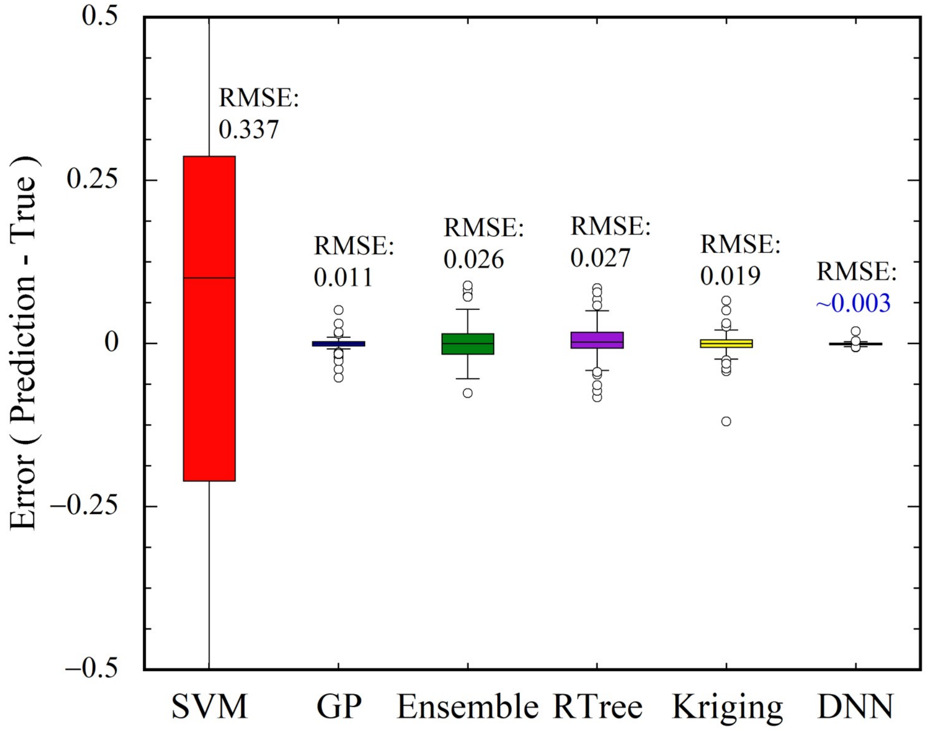

Figure S1. Root mean square error (RMSE) value comparison of various surrogate modelling methods. Validation error distributions of support vector machine (SVM), gaussian process (GP), ensemble learning (Ensemble), regression tree (RTree), Kriging method (Kriging)[1], and deep neural network (DNN) are plotted. As shown, DNN has the lowest RMSE value among the methods.

2 Global Sensitivity Analysis

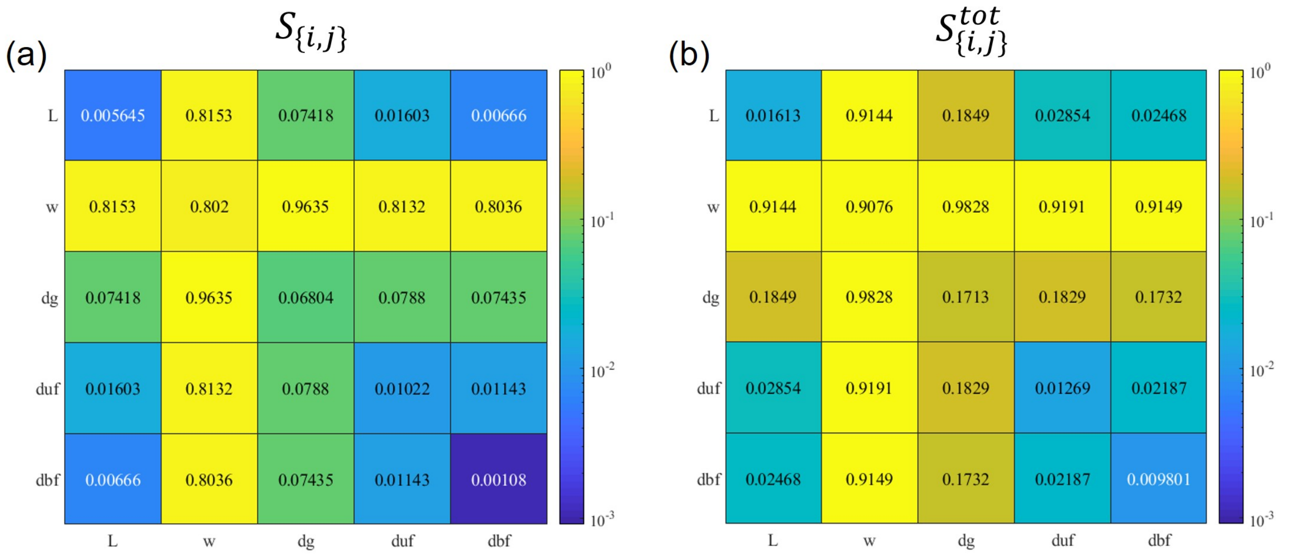

Figure S2. Global sensitivity of each design parameter is calculated using Monte Carlo estimation[2]. Each sensitivity index is illustrated as a heat-map chart with logarithmic heat scale. (a) $S_{\{i,j\}}$ is the sensitivity, related to only parameters i and j; (b) $S^{tot}_{\{i,j\}}$ is the total sensitivity, which is relevant to at least one of i or j. As shown, w and d_g are topmost and second most sensitive among the parameters.

3 Comparing number count of required RCWA computation

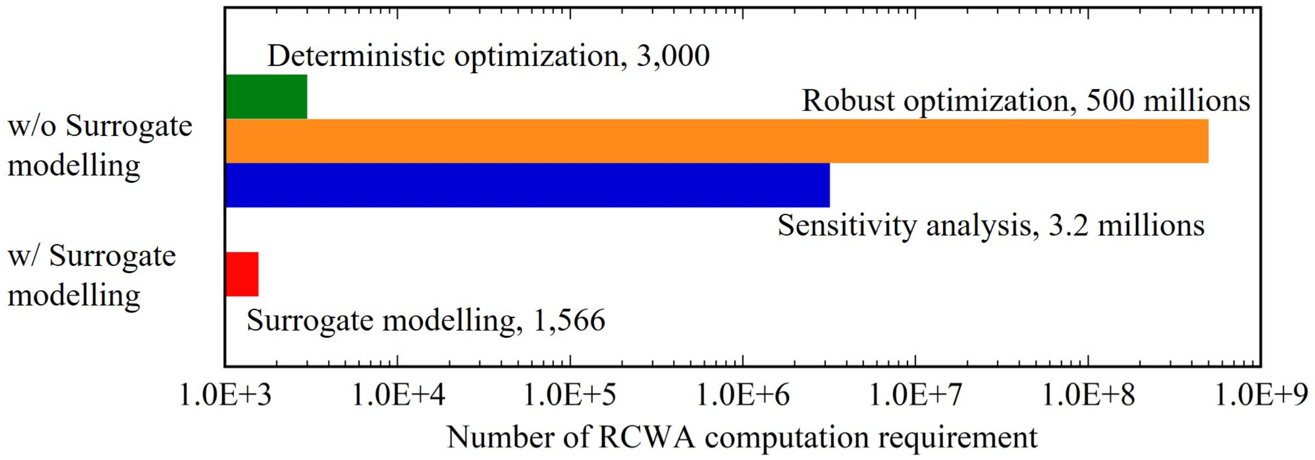

Figure S3. Number of actual RCWA computations required for deterministic and robust optimizations as well as for the sensitivity analysis without surrogate DNN modelling; and for surrogate DNN modelling. Note that one RCWA computation takes 20 min.

References

1. Choi, J., Kim, M., Kang, K., Lee, I. & Lee, B. J. Robust optimization of a tandem grating solar thermal absorber. *J. Quant. Spectrosc. Radiat. Transf.* **209**, 129–136 (2018).
2. Cannavó, F. Sensitivity analysis for volcanic source modeling quality assessment and model selection. *Comput. & Geosci.* **44**, 52–59 (2012).
